# Utility of Serum Procalcitonin and Its Clearance in Predicting Outcomes in COVID-19 Patients

**DOI:** 10.7759/cureus.60203

**Published:** 2024-05-13

**Authors:** Nazia Mehfooz, Tajamul Hussain Shah, Farhana Siraj, Syed Mudasir Qadri, Umar H Khan, Suhail Mantoo, Ajaz N Koul, Mushtaq Ahmad, Muzaffar Bindroo, Shaariq M Naqati

**Affiliations:** 1 Pulmonary Medicine, Sher-I-Kashmir Institute of Medical Sciences, Srinagar, IND; 2 Internal Medicine, Sher-I-Kashmir Institute of Medical Sciences, Srinagar, IND; 3 Rheumatology, Sher-I-Kashmir Institute of Medical Sciences, Srinagar, IND

**Keywords:** cytokine storm, acute hypoxic respiratory failure, community acquired pnemonia, procalcitonin, covid-19

## Abstract

Introduction

Identification of coronavirus disease 2019 (COVID-19) patients at risk of worse clinical outcomes is crucial to improving patient care. Various biochemical markers have been used to predict outcomes in such patients. We aimed to evaluate the role of serum PCT (procalcitonin) and the utility of PCT clearance (PCTc) in predicting the outcome of patients with COVID-19 illness.

Methods

We prospectively included 39 patients with severe or critical COVID-19 illness with an age equal to more than 18 years. In addition to routine baseline investigations, serum PCT was measured at admission (PCT1) and day 5 of hospitalization (PCT2). PCTc was calculated using the formula $(PCTc (\%) = (PCT1-PCT2/PCT1) x 100)$.

Results

We observed that serum PCT at admission was significantly higher in non-survivors (median: 1.9 ng/ml IQR: 0.51-4.23) compared to survivors (median 0.35 (IQR: 0.1-1.2), p 0.002). On serial serum-PCT estimation, non-survivors had persistently elevated serum-PCT (median PCT1:1.9 ng/ml (IQR: 0.51-4.23) to median PCT2: 1.9ng/ml (IQR: 0.83-2.72), p 0.51) than survivors (median PCT1:0.35ng/ml (IQR: 0.1-1.19) to median PCT2: 0.15ng/ml (IQR: 0.05-0.29), p 0.01). However, no difference in serum PCTc was observed between the two groups (median: 35.3% (IQR: 12.5-84.9) in survivors vs. 71.7% (33.3-91.7) in non-survivors, p = 0.165).

Conclusion

Serum PCT is a potential biochemical marker that could predict outcomes in COVID-19 patients. Measurement of serial serum PCT and estimation of PCT clearance may serve as better predictors than a single value; however, well-designed studies are required to identify the definite role of serum PCT in COVID-19 patients of varying severity.

## Introduction

Patients infected with severe acute respiratory syndrome coronavirus 2 (SARS-CoV-2) experience a wide range of clinical manifestations, ranging from asymptomatic disease to severe or critical coronavirus disease 2019 (COVID-19)-related illness [1]. Identification of subgroups of patients at high risk of disease progression or mortality may help in prioritizing patient care and improving clinical outcomes [2,3]. Several clinical and laboratory parameters have been used in patients infected with SARS-CoV-2, either individually or in the form of a risk-scoring system, to predict the severity of illness, disease progression, and mortality [4,5]. Various studies have evaluated the role of serum levels of pro-calcitonin (PCT) in predicting disease severity and mortality in such patients [6-8]. PCT synthesis is triggered by various toxins and cytokines like interleukin-6 (IL-6) or tumor necrosis factor-alpha (TNF-ɑ). A higher level of such cytokines in various hyper-inflammatory states, including COVID-19 infection, is associated with elevated serum PCT concentrations in the absence of bacterial infection [9,10]. Several studies have reported that the assessment of serial serum PCT and PCT clearance is a better predictor of disease severity and outcome [11,12]. Limited data is available on the outcome of patients with COVID-19 illness and persistently elevated serum PCT levels with reduced PCT clearance [13]. Therefore, we aimed to evaluate the utility of repeating serum PCT levels and the relationship between persistently elevated serum PCT and the outcome of patients with severe and critical COVID-19 infection.

## Materials and methods

This was a single-center prospective observational study that was performed at the Sher-I-Kashmir Institute of Medical Sciences (SKIMS), a tertiary care hospital in northern India. The hospital served as the referral center for the management of severe COVID-19-related illnesses during the COVID-19 pandemic. The study was conducted during the second wave of the COVID-19 pandemic (March 2021 to May 2021). The study was approved by the Institutional Ethics Committee at SKIMS, with application number 143/2021. Written informed consent was taken from all study participants.

The study included adults aged 18 years or older with severe to critical COVID-19 illness. The COVID-19 infection was confirmed by the presence of a positive nasopharyngeal reverse transcriptase polymerase chain reaction (RT-PCR) for SARS-CoV-2. Pregnant and lactating women, patients with recent surgery, trauma, proven bacterial sepsis, and renal failure were excluded. In addition, patients with an expected survival of less than 24 hours were also excluded from the study.

The severity of COVID-19 was defined as per the WHO classification for COVID-19 disease severity. A severe COVID-19 illness was defined when any of the following criteria were met: (i) respiratory rate (RR) of more than or equal to 30 breaths/min; (ii) oxygen saturation (SPO2) less than or equal to 93% in the resting state; or (iii) PaO2/FiO2 (partial pressure of oxygen/fraction of inspired oxygen) less than or equal to 300 mmHg. A critical COVID-19 illness was defined by any of the following conditions: (i) respiratory failure requiring mechanical ventilation, (ii) shock, or (iii) other organ failure requiring ICU admission for monitoring and treatment.

Routine baseline investigations, including a complete blood count (CBC), kidney function test (KFT), liver function test (LFT), electrocardiogram (ECG), and chest radiography, were obtained from all patients included in the study. In addition, serum PCT concentration was assessed on day 1 of admission (PCT1) and repeated on day 5 of hospitalization (PCT2). Serum samples were stored at -80°C until analyzed. PCT estimation was done using a latex-enhanced immunoturbidimetric assay and a biochemical analyzer from Beckman Coulter Diagnostics (USA). It measures PCT within the range of 0.20-52 ng/mL. The clearance of PCT (PCTc) at day 5 was calculated as:$(PCTc (\%) = (PCT1-PCT2/PCT1) x 100)$. Positive clearance values indicate a decrease in the concentration of PCT, whereas negative clearance values indicate an increase in PCT concentration.

Information regarding various demographic features, co-morbid illnesses, severity of illness, laboratory parameters, length of hospital stay, and clinical outcome was noted by all included patients. Patients were managed in COVID-19-dedicated areas, including high-dependency units (HDUs) and intensive care units (ICUs). Patients received standards of care based on national and international guidelines and were modified if required as per the discretion of treating physicians.

Statistical analysis 

The continuous variables were presented as mean ± SD or median. Categorical variables were presented as numbers and percentages. To compare groups, we used the Pearson Chi-square (χ2) or Fisher's exact test for categorical variables and the independent samples t-test or Mann-Whitney U test for continuous variables, depending on their distribution. The receiver operating curve was used to determine the optimal serum procalcitonin cut-off value for predicting mortality. The clearance of PCT (PCTc) at day 5 was calculated as:$(PCTc (\%) = (PCT1-PCT2/PCT1) x 100)$.

## Results

A total of 39 patients with severe and critical COVID-19 illnesses were included in the study. The mean age of the study population was 57.7±16.5 years, with 53.8% of them being males (Table 1).

**Table 1:** Demographic characteristics and severity of illness of study patients

Sociodemographic data			Total (n=39)	Survivors (n=28)	Non-survivors (n=11)	p-value
Age, mean (±std) in years			57.72±16.48	58.21±17.2	56.45±15.2	0.757
Gender n (%)		Male	21 (53.8)	14 (50)	7 (63.6)	0.442
		Female	18 (46.2)	14 (50)	4 (36.4)	
Residence n (%)		Urban	21 (53.8)	16 (57.1)	5 (45.5)	0.51
		Rural	18 (46.2)	12 (42.9)	6 (54.5)	
Previous COVID-19 illness, n(%)			4 (10.2)	4 (14.3)	0 (0)	0.309
Severity of illness, n (%)	Severe		26 (66.6)	22 (78.5)	4 (36.3)	0.01
	Critical		13 (33.3)	6 (21.5)	7 (63.6)	

Out of a total of 39 patients, 66.7% (n = 26) had severe, whereas 33.3% (n = 13), had critical COVID-19-related illnesses. In total, 11 (28.2%) patients died during hospitalization, and 28 (71.8%) patients were discharged. The median duration of hospital stay was 9.5 days (IQR 6-12) in survivors and 16 days (IQR 7-21) in non-survivors (p = 0.063). There was no significant difference between the survivors and non-survivors with respect to demographic characteristics and co-morbidities (Table 1). The majority of the patients who died had a critical COVID-19-related illness (63.6%, n = 7), while 36.4% (n = 4) had a severe illness. The most common co-morbid illness identified was hypertension (56.4%, n = 22), followed by diabetes mellitus (43.6%, n = 17) and chronic kidney disease (12.8%, n = 5) (Table 2).

**Table 2:** Co-morbid illnesses of study patients

Comorbidity n (%)	Total (n=39)	Survivors (n=28)	Non-Survivors (n=11)	p-value
Hypertension	22(56.4)	16(57.1)	6(54.5)	0.883
Diabetes	17(43.5)	11(39.3)	6(54.5)	0.387
Chronic Kidney Disease	5(12.8)	4(14.3)	1(9.1)	0.662
No comorbidity	11(28.2)	9(32.1)	2(18.2)	0.383

Serum levels of pro-calcitonin were assessed at admission (PCT1) and were repeated on day 5 (PCT2). Repeat measurements were available for 35 patients, as four patients died within five days of hospitalization. Serum PCT concentration at admission didn’t differ between severe and critically ill COVID-19 patients (median 0.52 (IQR: 0.22-1.34)) in severe vs. median 0.52 (IQR: 0.23-1.31) in critical, p = 0.78) (Table 3).

**Table 3:** Comparison of Serum PCT in patients with severe and critical COVID-19 illness PCT, Procalcitonin

Procalcitonin	Severe (n=26)	Critical (n=13)	p-value
Serum PCT ng/ml on admission, Median (IQR)	0.52(0.22-1.34)	0.52(0.23-1.31)	0.78

Compared to survivors, non-survivors had significantly higher serum concentrations of PCT1 ( median: 0.35 (0.1-1.2) vs. 1.9 ng/ml (IQR: 0.51-4.23), p 0.002) and PCT2 (median: 0.15 (0.05-0.29) vs 1.9 ng/ml (IQR: 0.83-2.7), p 0.001) (Table 4). Similarly, serum lactate dehydrogenase (LDH) and D-dimer levels were significantly higher in non-survivors compared to survivors (Table 4). However, only serum LDH showed a significant positive correlation with PCT1 and PCT2. (r = 0.541, p = 0.01 for PCT1, and r = 0.516, p = 0.01 for PCT2).

**Table 4:** Laboratory profile of survivors and non survivors Hb, hemoglobin; TLC, total leucocyte count; NLR, neutrophil-to-lymphocyte ratio; LDH, lactate dehydrogenase; CPK, creatine phosphokinase; IL-6, interleukin 6; PCT1, procalcitonin on Day 1; PCT2, procalcitonin on Day 5.

Laboratory parameters	Survivors (n=28)	Non-survivors (n=11)	p-value
Hb gm/dl (Mean±SD)	12.3±2.9	11.2±2.5	0.27
TLC *10^9^/L (Mean±SD)	7.38±3.7	11.9±9.5	0.15
NLR (IQR)	7.1(2.9-13.59)	11.25(3.11-22.5)	0.36
Creatinine median (IQR)	1.09(0.74-1.5)	1.3(0.87-1.71)	0.26
Albumin gm/L (Mean±SD)	3.3(2.94-3.63)	3.17(2.5-3.7)	0.31
Lactate mmol/L median (IQR)	1.73(1.22-2.17)	2.1(1.58-2.5)	0.31
D-dimer ng/ml median (IQR)	350.5(119.5-660)	778(453-1300)	0.02
LDH units/L median (IQR)	299.5(131.75-424.25)	421(225-783)	0.04
CPK units/L median (IQR)	202(83.75-273.5)	147(87-207)	0.81
IL-6 pg/ml median (IQR)	25.6(11.56-53)	45(22-102)	0.16
PCT1 ng/ml median (IQR)	0.35(0.1-1.19)	1.9(0.51-4.23)	0.002
PCT2 ng/ml median (IQR)	0.15(0.05-0.29)	1.9(0.83-2.7)	0.001

It was also noted that survivors had a significant reduction in serum pro-calcitonin concentration on serial measurement (median PCT1:0.35 ng/ml (IQR: 0.1-1.19) to median PCT2: 0.15 ng/ml (IQR: 0.05-0.29), p 0.01) in survivors and (median PCT1:1.9 ng/ml (IQR: 0.51-4.23) to median PCT2: 1.9 ng/ml (IQR: 0.83-2.72) in non-survivors, p 0.51). In other words, non-survivors had persistently elevated serum PCT levels as compared to survivors (Table 5).

**Table 5:** Comparison between the admission day and day 5 serum PCT in survivors and non survivors PCT1, procalcitonin on day 1; PCT2, procalcitonin on day 5

Outcome	PCT1	PCT2	p-value
Survivors	0.35(0.1-1.19)	0.15(0.05-0.29)	0.01
Non-survivors	1.9(0.51-4.23)	1.9(0.83-2.72)	0.51

As regards PCT clearance, no significant difference was observed between survivors and non-survivors [median: 35.3% (IQR: 12.5-84.9) in survivors vs. 71.7% (33.3-91.7) in non-survivors, p = 0.165 (Table 6).

**Table 6:** Serum procalcitonin clearance between survivors and non-survivors PCT, procalcitonin

Serum Procalcitonin	Survivors	Non-survivors	p-value
PCT clearance, median (IQR)	35.3% (12.5-84.9)	71.7% (33.3-91.7)	0.165

The optimal cut-off values of PCT1 and PCT2 that predicted mortality were 1.2 ng/mL and 0.44 ng/mL, respectively. The area under curve (AUC) of the receiver operating characteristic (ROC) analysis for PCT1 and PCT2 in predicting hospital mortality was 0.82 and 0.89, with a sensitivity of 72.7%/88.9% and a specificity of 78.6%/85.7%, respectively (Table 7).

**Table 7:** Optimal cut-off, AUC, sensitivity and specificity of PCT1 and PCT2 in predicting mortality using Youden’s index AUC, Area under curve; PCT1, procalcitonin on day 1; PCT2, procalcitonin on day 5

Parameter	Cut-off value	AUC	Sensitivity (%)	Specificity (%)
PCT1	1.2 ng/ml	0.82	72.7	78.6
PCT2	0.44 ng/ml	0.89	88.9	85.7

## Discussion

This single-center prospective observational cohort study including severe to critically ill COVID-19 patients demonstrated that on admission, serum PCT did not differ significantly between patients with severe and critical COVID-19 illness. Non-survivors of severe to critical COVID-19 had persistently higher serum PCT as compared to survivors; however, no significant difference was noted in terms of serum PCT clearance between the two groups.

Several studies have reported that serum PCT at admission is associated with the severity of the COVID-19 illness. In a meta-analysis of four studies that investigated the role of serum PCT in distinguishing patients with or without severe COVID-19, the pooled odds ratio showed that increased serum PCT values were associated with a fivefold increased risk for severe SARS-CoV-2 infection [14]. Similarly, Tong-Minh et al. in a retrospective analysis of 332 (n = 105) COVID-19 patients, reported that elevated serum PCT levels were associated with severe COVID-19 infection after adjustment for bacterial co-infection [15]. In our study, although the serum PCT at admission was elevated, no significant difference was observed between severe and critical COVID-19 illnesses. A smaller sample size, a disproportionate number of patients in two groups, and the heterogeneity in defining the severity of disease by various authors could explain our results. In addition, it may be presumed that serum PCT could differentiate non-severe from severe illness and not severe from critical related to COVID-19.

Serum PCT levels have been used to predict outcomes in terms of mortality in patients with bacterial or viral illnesses. We reported significantly higher mortality in patients with severe to critical COVID-19 and elevated serum PCT levels on admission. Similar to our findings, in a retrospective review of 271 critically ill COVID-19 patients, elevated serum PCT on admission was strongly associated with mortality (OR 5.65; 95% CI: 2.14-14.9) [16].

We reported that a serum PCT cut-off value of 1.2 ng/ml on admission and 0.44 ng/ml on day 5 of hospitalization predicted mortality with a sensitivity of 72.7% and 88.9% and a specificity of 78.6% and 85.7%, respectively. Jeyapalina et al. [6] reported serum PCT greater than 0.20 ng/ml to be independently associated with mechanical ventilation and mortality in hospitalized COVID-19 patients (unadjusted HR, 2.28, 95%CI: 2.16-2.41 for mortality). In a meta-analysis that included patients of various severities (non-severe and severe), the cut-off value that predicted mortality ranged from 0.05 to 0.5 ng/mL [17]. Higher values of serum PCT that predicted mortality in our study could be likely due to the inclusion of only severe to critical COVID-19.

We noticed persistently elevated levels of serum PCT on serial measurements in patients with severe to critical COVID-19 illnesses strongly associated with in-hospital death. In accordance with our results, a prospective multicenter study of 201 COVID-19 patients with respiratory involvement who require admission to the ICU found elevated serum PCT during hospital stay (day 3) significantly predicted mortality [18]. However, it is to be noted that serum PCT on the day of admission to the ICU didn’t predict mortality. Similarly, Hu et al. [7] analyzed 95 COVID-19 patients with a severity of illness ranging from moderate to critical (62 moderate, 21 severe, and 12 critical). Out of 93 patients, six died during their hospital stay (all belonged to critical COVID-19). It was observed that elevated serum PCT levels on serial measurements significantly predicted mortality. These results highlight that serial measurement of serum PCT in COVID-19 patients may be useful in identifying the subgroup of patients at high risk of mortality and, therefore, may help in improving management.

Several studies evaluated the role of serum PCT clearance in predicting mortality in patients with bacterial sepsis and septic shock [12,19]. These studies observed that PCT clearance is a better predictor of mortality than a single PCT cut-off value. In a larger multicenter observational study that included patients with sepsis (n = 858), serum PCT was measured daily for five days [11]. A subgroup of sepsis patients with persistently elevated serum PCT at day 4 and non-resolution of serum PCT by 80% had significantly higher 28-day all-cause mortality. However, in a retrospective study by Nassar et al. [20] that included patients with cancer and sepsis, procalcitonin clearance at 24, 48, 72, and 96 hours did not accurately predict hospital mortality and 90-day mortality rate. In an attempt to evaluate the utility of serum PCT and its clearance in predicting outcomes in patients with COVID-19 illness Taha et al. [13] prospectively studied 63 patients infected with SARS-CoV-2 (n = 32, 50.8% non-severe, and n = 31, 49.2% severe). They observed that serum PCT on admission, day 3, and day 5 was significantly higher in non-survivors. As regards serum PCT clearance, non-survivors had significantly reduced clearance as compared to survivors (median: 50.6% (IQR: 769.15-19.53) vs. 25% (13.39-42.82); p = 0.003, and median: ˗ 325.7% (IQR: 1919.61-96.13) vs. 51.56% (34.37-78.95); p ≤ 0.001, respectively). The persistence of PCT in COVID-19 could be caused by either elevated cytokine levels (a cytokine storm) or an associated secondary bacterial infection, both of which can lead to worsening organ failure and death [21]. Despite the persistently elevated serum PCT level at day 5 of admission in non-survivors, our study did not demonstrate the role of serum PCT clearance in predicting mortality in patients with severe to critical COVID-19 illness. The inclusion of only severe or critical COVID-19 patients and a smaller sample size could be postulated as a possible explanation for our results.

We pointed out that, in addition to elevated levels of serum PCT, other inflammatory markers, including serum LDH and D-dimer levels, were significantly higher in non-survivors of severe to critical COVID-19 patients as compared to survivors. In a prospective observational study of 180 severe COVID-19 patients, levels of neutrophil percentage, C-reactive protein (CRP), IL-6, PCT, and LDH were significantly higher in non-survivors [22]. The authors also observed that IL-6 and LDH were the most sensitive and specific markers for predicting mortality among severe COVID-19 patients. Asghar et al. showed that the neutrophil-to-lymphocyte ratio (NLR) significantly predicted days of ICU stay, the need for invasive ventilation, and death in COVID-19 patients. NLR also showed a good correlation with other biochemical markers, including CRP (C-reactive protein), PCT, and D-dimer [23].

Our study has a few limitations. First, it was a single-center observational study that included only severe to critically ill COVID-19 patients; therefore, the results may not be applicable to all COVID-19 patients. Second, the sample size was small (due to inclusion criteria), and the distribution of cases into study groups (survivors and non-survivors) was disproportionate. Third, information regarding microbiological data was incomplete.

## Conclusions

This study reported that mortality was significantly higher in the subgroup of severe to critical COVID-19 patients with elevated serum PCT on admission and in those with persistently elevated serum PCT. In patients with low PCT clearance, the mortality difference was not statistically significant. Well-designed multicenter randomized controlled trials are required to evaluate the role of PCT clearance in predicting the outcome of patients with COVID-19-related illnesses.
